# Highly efficient radiosensitization of human glioblastoma and lung cancer cells by a G-quadruplex DNA binding compound

**DOI:** 10.1038/srep16255

**Published:** 2015-11-06

**Authors:** Patrick Merle, Marine Gueugneau, Marie-Paule Teulade-Fichou, Mélanie Müller-Barthélémy, Simon Amiard, Emmanuel Chautard, Corinne Guetta, Véronique Dedieu, Yves Communal, Jean-Louis Mergny, Maria Gallego, Charles White, Pierre Verrelle, Andreï Tchirkov

**Affiliations:** 1 Clermont Université, Université d’Auvergne, EA 7283, 63003 Clermont-Ferrand, France; 2 CHU Clermont-Ferrand, Service de Pneumologie Oncologie Thoracique, 63003 Clermont-Ferrand, France; 3 Institut Curie, UMR 176, Centre Universitaire Paris-Sud, 91405 Orsay, France; 4 Génétique, Reproduction et Développement, UMR CNRS 6293, Clermont Université, INSERM U1103, 63170 Aubière, France; 5 Centre Jean Perrin, Département de Radiothérapie, 63011 Clermont-Ferrand, France; 6 Centre Jean Perrin, Laboratoire OncoGènAuvergne, 63011 Clermont-Ferrand, France; 7 Université de Bordeaux, 33076 Bordeaux, France; 8 INSERM U1212, ARNA laboratory, IECB, 33607 Pessac, France; 9 Département de Radiothérapie, Institut Curie, Paris, France; 10 Clermont Université, Université d’Auvergne, Histologie Embryologie Cytogénétique, 63001 Clermont-Ferrand, France; 11 CHU Clermont-Ferrand, Service de Cytogénétique Médicale, 63003 Clermont-Ferrand, France

## Abstract

Telomeres are nucleoprotein structures at the end of chromosomes which stabilize and protect them from nucleotidic degradation and end-to-end fusions. The G-rich telomeric single-stranded DNA overhang can adopt a four-stranded G-quadruplex DNA structure (G4). Stabilization of the G4 structure by binding of small molecule ligands enhances radiosensitivity of tumor cells, and this combined treatment represents a novel anticancer approach. We studied the effect of the platinum-derived G4-ligand, Pt-ctpy, in association with radiation on human glioblastoma (SF763 and SF767) and non-small cell lung cancer (A549 and H1299) cells *in vitro* and *in vivo*. Treatments with submicromolar concentrations of Pt-ctpy inhibited tumor proliferation *in vitro* with cell cycle alterations and induction of apoptosis. Non-toxic concentrations of the ligand were then combined with ionizing radiation. Pt-ctpy radiosensitized all cell lines with dose-enhancement factors between 1.32 and 1.77. The combined treatment led to increased DNA breaks. Furthermore, a significant radiosensitizing effect of Pt-ctpy in mice xenografted with glioblastoma SF763 cells was shown by delayed tumor growth and improved survival. Pt-ctpy can act in synergy with radiation for efficient killing of cancer cells at concentrations at which it has no obvious toxicity *per se*, opening perspectives for future therapeutic applications.

Radiochemotherapy has become a standard treatment for localized advanced tumors. Radiotherapy with concomitant temozolomide is used in treatment of glioblastoma multiforme (GBM)^1^. In non-small cell lung cancer (NSCLC), concomitant platinum-based chemotherapy and radiotherapy is the standard treatment for locally advanced stage III disease^2^. However, intrinsic radioresistance ultimately leads to tumor recurrence within the radiation fields; therefore increasing the radiosensitivity of GBM and NSCLC cells would improve patient outcome. Chemical modulators of DNA damage response and apoptosis represent potential radiosensitizers^3^. In addition, telomeric dysfunction due to genetic deficiency in telomerase activity has been shown to increase radiosensitivity and to decrease the capacity of DNA repair^4^.

Telomeres, the nucleoprotein structures that protect the end of chromosomes, are crucial for maintaining the stability and the integrity of the genome^5^. Telomeric DNA is capped by Shelterin complex proteins and telomere length is tightly regulated by telomerase, a ribonucleoprotein enzyme complex with reverse transcriptase activity which adds hexameric GGTTAG repeats to the chromosomal ends^6^. Telomerase is overexpressed in more than 85% of human cancers and contributes to unlimited proliferation of tumor cells^7^. Therefore, targeting telomeres and/or telomerase by small molecules is a promising strategy for cancer treatment^8^.

In this context, we have been interested in targeting of DNA secondary structures called G-quadruplexes, which are formed in G-rich regions such as telomeres^9^. The stabilization of these structures using small molecule ligands may induce a strong DNA damage response at telomeres^10^. Quadruplex-targeting molecules (quadruplex ligands or G4-ligands) cause telomere uncapping by displacement of protective proteins and affect telomerase function^10^,^11^,^12^,^13^,^14^,^15^.

We previously validated this strategy *in vitro* with a first-generation G4-ligand TAC that radiosensitized human GBM cells^16^. Here, we present an analysis of a second-generation G4-ligand Pt-ctpy that strongly enhances the sensitivity of human GBM and NSCLC cells to ionizing radiation both *in vitro* and *in vivo*.

## Results

### Pt-ctpy inhibits GBM and NSCLC cell growth

**Figure 1:**
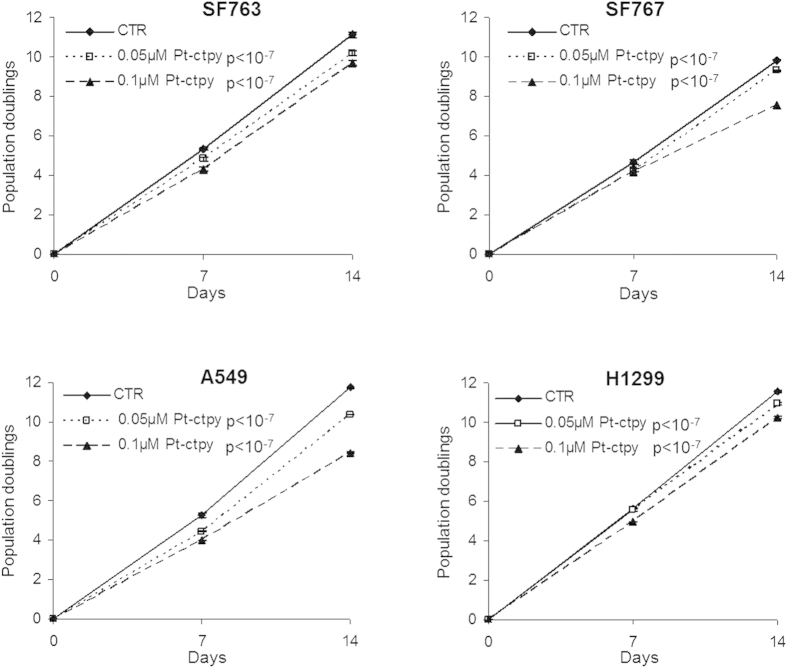
Effects of Pt-ctpy on proliferation of GBM (SF763, SF767) and NSCLC cells lines (A549, H1299). Cells were treated continuously with Pt-ctpy (0.05 and 0.1 μM) for 14 days and compared with non-treated cells (NT). Population doublings of the cell lines are shown. Mean ± SE values of 3 independent experiments, each performed in triplicate, are presented. Pt-ctpy induced a significant dose-dependent proliferation inhibition in both cell lines (ANOVA).

**Figure 2:**
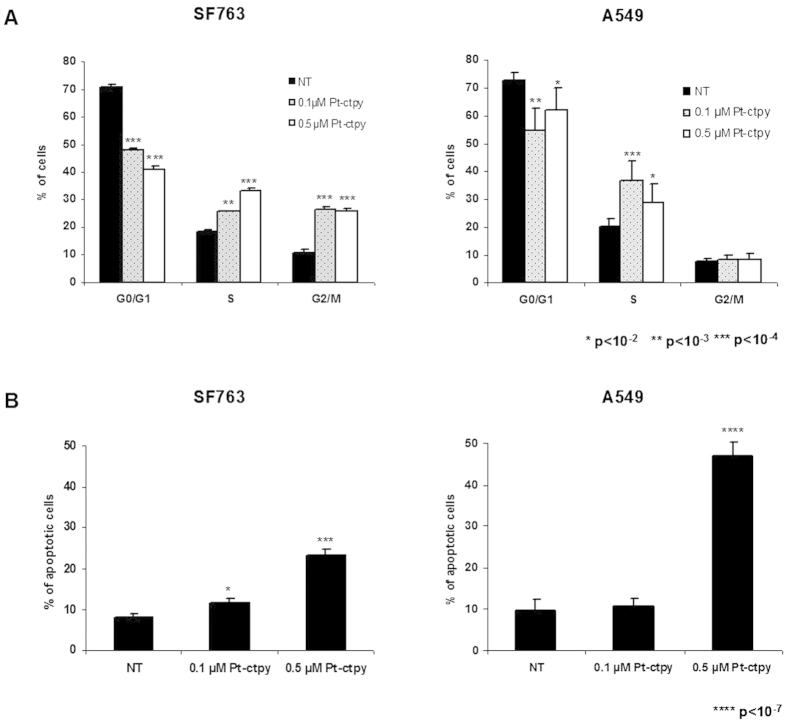
Effects of Pt-ctpy on cell cycle and apoptosis. (**A**) Cell cycle analysis: distribution of cells in the different cell cycle phases. SF763 and A549 cells exposed to Pt-ctpy showed significant modifications in this distribution. (**B**) Flow cytometry assessment of sub-G0/G1 fraction, corresponding to apoptotic cells. Tumor cells treated with Pt-ctpy exhibited a significant increase in Sub-G0/G1 population (*t*-test).

GBM cell lines (SF763 and SF767) and NSCLC cells lines (A549 and H1299) were treated for 14 days with 0.05 and 0.1 μM of Pt-ctpy. This treatment inhibited the growth of all cell lines in a concentration-dependent manner (*p* < 10^−7^, Fig.1), while trypan blue staining indicated no toxicity (<10% of cells stained; not shown). In parallel, we tested the same concentrations of Pt-ctpy on control normal human fibroblasts. No changes in proliferation were observed (Supplementary Fig. S1). After 14 days of treatment, cell distribution among different phases of the cell cycle was studied using flow cytometry in SF763 and A549 cells. We observed (*i*) a significant decrease of cells in the G0/G1 phase, (*ii*) a significant accumulation of cells in the S-phase and *(iii)* an accumulation of SF763 cells in the G2/M-phase (Fig. 2A). To assess apoptosis, the sub-G0/G1 cell cycle fraction was evaluated in both cell lines by flow cytometry after 14 days of treatment with Pt-ctpy (0.1 and 0.5 μM). The apoptotic sub-G0/G1 fraction was low in untreated controls (<10%). In contrast, the sub-G0/G1 fraction increased to 23% in SF763 cells and 47% in A549 cells (*p* < 10^−4^ and *p* < 10^−7^) after 0.5 μM Pt-ctpy treatment (Fig. 2B).

### Pt-ctpy triggers *hTERT* overexpression and *TRF1* underexpression

**Figure 3:**
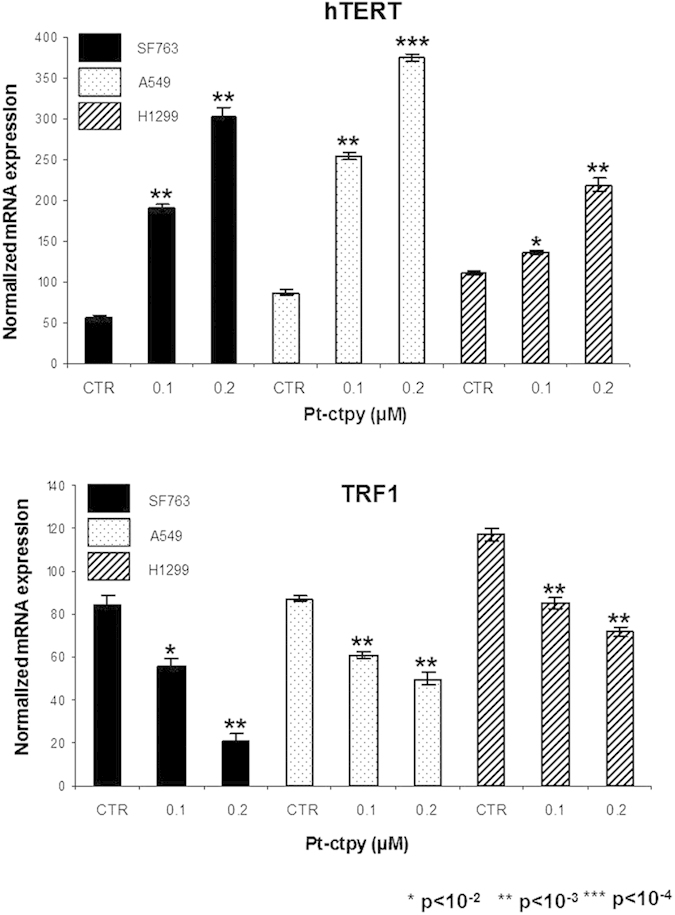
Pt-ctpy effects on *hTERT* and *TRF1 mRNA* expression levels. Quantitative real-time RT-PCR results for the expression of *hTERT* and telomere-related genes *TRF1.* In both cell lines, the treatment with Pt-ctpy led to an increase in the expression of *hTERT* (**A**), whereas the expression of *TRF1* (**B**) was decreased after Pt-ctpy treatment (*t*-test).

We evaluated the expression levels of *hTERT* (the catalytic subunit of telomerase) and *TRF1* (one of the core members of the shelterin complex) genes in GBM and NSCLC cells treated with Pt-ctpy for 7 days (Fig. 3A). In comparison with untreated controls, the expression of *hTERT* was significantly increased (6-fold for SF763, 5-fold for A549, and 2-fold for H1299 cells) (*p* < 10^−3^), and *TRF1* expression was decreased (4-fold for SF763, 1.7-fold for A549, and 1.6-fold for H1299 cells) after Pt-ctpy treatment (*p* < 10^−3^) (Fig. 3A).

### Pt-ctpy radiosensitizes GBM and lung cancer cells *in vitro*

The intrinsic radiosensitivities of GBM and NSCLC cell lines were studied using standard clonogenic survival assay after irradiation with increasing doses (2, 4, 6 and 8 Gy). GBM and NSCLC lines were found to be relatively radioresistant with a survival fraction at 2 Gy of 80.6% for SF763, 59.3% for SF767, 66.8% for A549 and 71.6% for H1299 cells.

**Figure 4:**
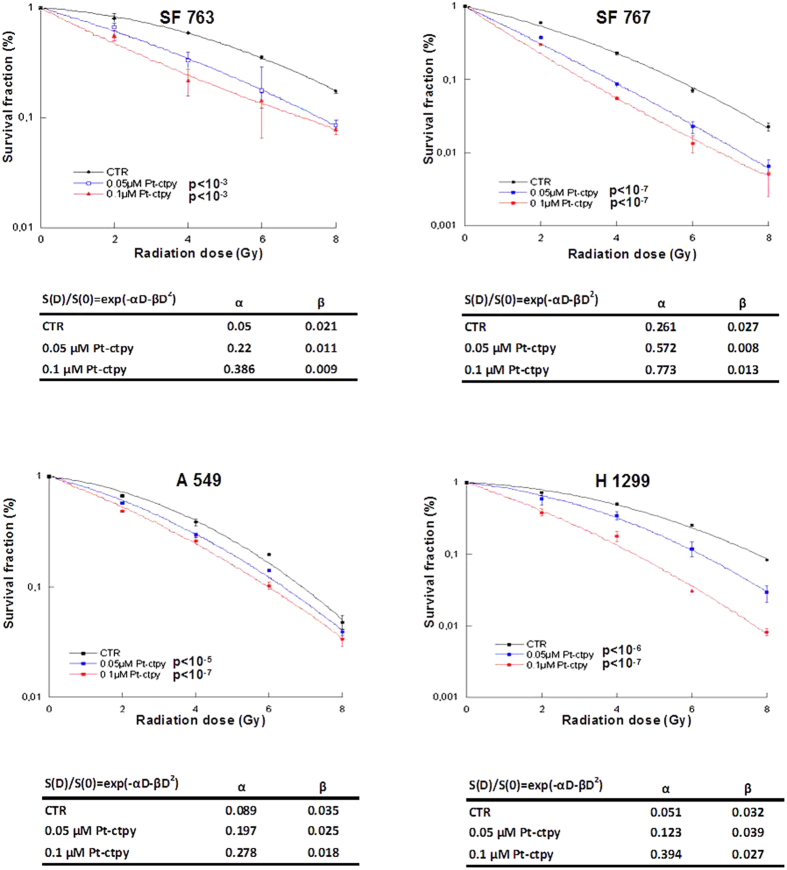
Pt-ctpy radiosensitizes GBM and NSCLC cells. After continuous treatment with 0.05 μM and 0.1 μM Pt-ctpy for one week and/or X-ray irradiation with doses ranging from 2 to 8 Gy, survival of GBM and NSCLC were determined using colony formation assay. Mean ± SE values of 3 independent experiments each performed in triplicate are shown. Survival (S) data after a radiation dose (D) were fit according to the linear-quadratic model. The linear parameter α and the quadratic parameter β are given for each experimental condition. Radiation-induced killing of Pt-ctpy treated cells was significantly enhanced in a concentration-dependent manner (ANOVA).

We then combined Pt-ctpy treatment with radiation. GBM or NSCLC cells were treated with 0.05 and 0.1 μM Pt-ctpy for one week. These cells were then irradiated with single doses ranging from 0 to 8 Gy. The comparison between clonogenic survival curves after either irradiation alone or combined treatment demonstrated a strong, concentration-dependent enhancement of radiation sensitivity after Pt-ctpy treatment (Fig. 4). Survival (S) data after irradiation were fitted according to the linear-quadratic model: S(D)/S(0) = exp(– αD – βD^2^). The α and β parameters were used to calculate the dose enhancement factor at 10% survival (DEF_10_). The DEF_10_ values for 0.05 and 0.1 μM Pt-ctpy were 1.24 and 1.32, respectively in SF763 cells; 1.46 and 1.77 in SF767 cells; 1.08 and 1.15 in A549 cells; 1.25 and 1.75 in H1299 cells.

**Figure 5:**
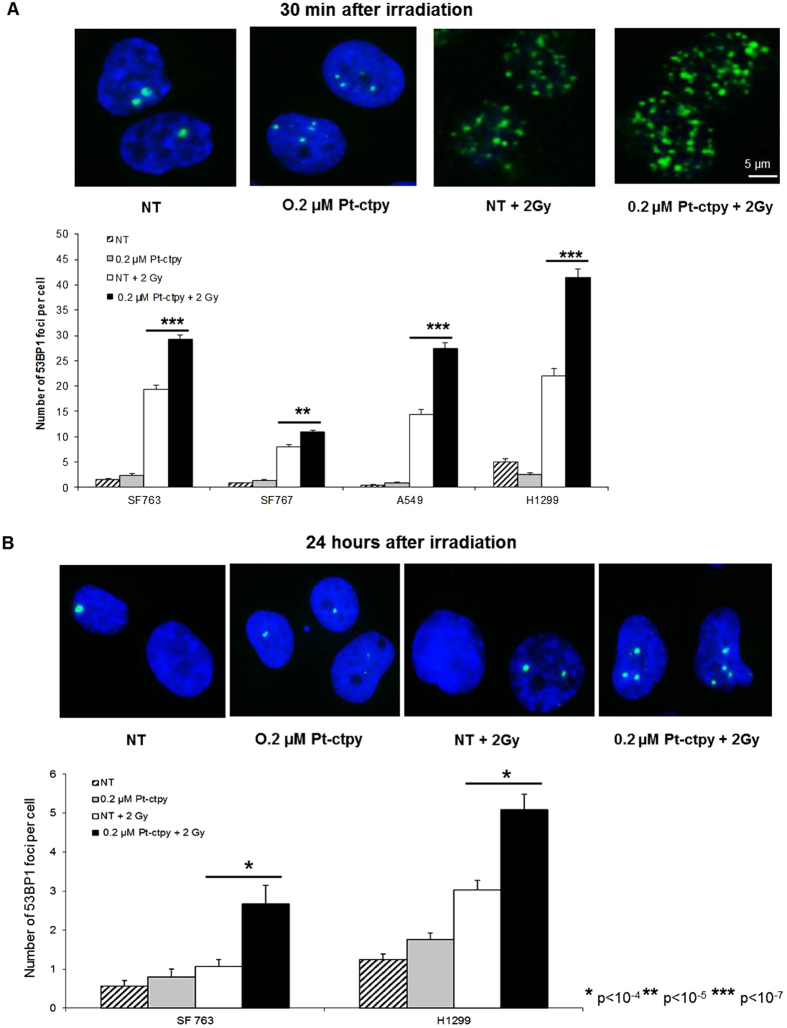
Induced and residual DNA damage in GBM or NSCLC cells treated with radiation and Pt-ctpy. Results of 53BP1 immunofluorescence analysis of GBM (SF763, SF767) and NSCLC (A549, H1299) cells pre-treated by Pt-ctpy during 24 hours and irradiated with 2 Gy. Cells were fixed 0.5 h (**A**) or 24 h (**B**) after irradiation. (**A**) Representative images of SF763 cell nuclei fixed 0.5 h following irradiation stained with 4′,6-diamidino-2-phenylindole (DAPI, blue) and 53BP1 (green) exposed to Pt-ctpy and/or irradiation. The increase of 53BP1 foci number per cell was significant in GBM or NSCLC cells exposed to 0.2 μM Pt-ctpy and radiation compared with non-treated (NT) cells and cells treated with radiation alone (H-test). (**B**) Representative images of SF763 cell nuclei fixed 24 h after irradiation stained with 4′,6-diamidino-2-phenylindole (DAPI, blue) and 53BP1 (green) after exposure to Pt-ctpy and/or irradiation. The number of residual DSB 24 h after irradiation was significantly increased after combined treatment (H-Test).

To investigate possible mechanisms underlying this radiosensitizing effect, we evaluated the induction of DNA double-stranded breaks (DSB) by detecting 53BP1, a protein that co-localizes with DSB, 0.5 h after irradiation with 2Gy as well as residual DSB levels 24 h following irradiation in tumor cells treated or not with 0.2 μM Pt-ctpy. Remarkably, while Pt-ctpy alone did not induce a significant increase in the number of foci, we observed a substantial increase in the number of 53BP1 foci at 0.5 h after irradiation in combined treatment as compared with irradiation alone (Fig. 5A). This striking sensitizing effect (up to 30–40%) was more or less similar for all cell lines, although the GBM SF767 cell line appeared to be less sensitive. The level of residual (unrepaired) DSB 24 hours after irradiation was also higher after combined treatment (Fig. 5B).

**Figure 6:**
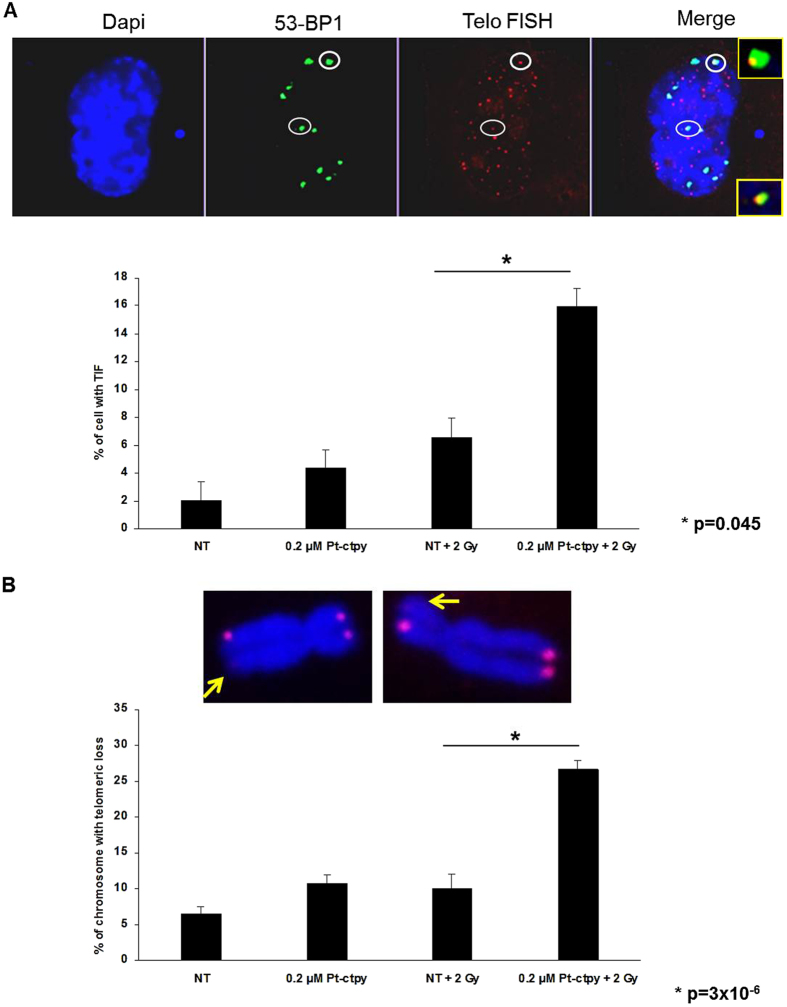
Telomere damage persists in NSCLC cells treated with radiation and Pt-ctpy. Results of 53BP1 immunofluorescence analysis, telomere FISH and telomeric loss of NSCLC (H1299) cells treated with Pt-ctpy for 24 h and then irradiated with 2 Gy. Cells were fixed 24 h after irradiation. (**A**) Representative images of H1299 cell nuclei stained with 4′,6-diamidino-2-phenylindole (DAPI, blue), 53BP1 (green), telomere FISH (red) exposed to Pt-ctpy and/or irradiation. The increase in the number of TIF was significant in NSCLC cells exposed to 0.2 μM Pt-ctpy and radiation versus non treated (NT) and radiation (*t*-test). (**B**) Representative images of H1299 metaphase chromosomes stained with 4′,6-diamidino-2-phenylindole (DAPI, blue) and telomere FISH (red) from cells exposed to Pt-ctpy and/or irradiation. After combined treatment, we observed a significant increase in the number of complete telomere loss (*t*-test).

Telomeres are G-rich regions particularly prone to G-quadruplex formation. When fixed by Pt-ctpy binding, this secondary structure may block replication forks and thus leads to loss of telomeric repeats. These dysfunctional telomeres will then be recognized as double strand breaks. We quantified telomere dysfunction-induced foci (TIFs) through combined immunolocalization of 53BP1 and telomeric DNA FISH (Fluorescent *In Situ* Hybridization). These analyses showed a significant increase in the number of TIF 24 h post-irradiation, after combined treatment (Fig. 6A). In agreement with this observation, telomeric FISH analysis of metaphase spreads at the same time-point revealed that complete telomere loss was significantly more frequent after the combination Pt-ctpy treatment and radiation, than after radiation alone (Fig. 6B).

In addition to its quadruplex-binding properties, Pt-ctpy is a monofunctional platinum complex and can form metal coordination adducts at the level of quadruplex DNA or, eventually, duplex DNA^17^,^18^. It was thus important to compare the effect of Pt-ctpy with platinum-based chemotherapy drugs, which are known to act as radiosensitizing agents in a number of cancers^19^. Using cisplatin in the same concentration conditions (0.2 μM), we found no effect on GBM and NSCLC cells in terms of growth inhibition and radiosensitization (data not shown). This indicates that the radiosensitizing effect of Pt-ctpy is due to specific properties of this compound and does not result from a classical DNA platination effect.

### Pt-ctpy radiosensitizes human GBM xenografts

We evaluated the radiosensitizing effect of Pt-ctpy in nude mice xenografted with SF763 tumor cells. Pt-ctpy treatment was given daily intra- and peritumoraly at 2 mg/kg/d. Pt-ctpy treatment was well tolerated. No toxic death or body weight losses were observed during treatment or in control mice without GBM xenografts.

**Figure 7:**
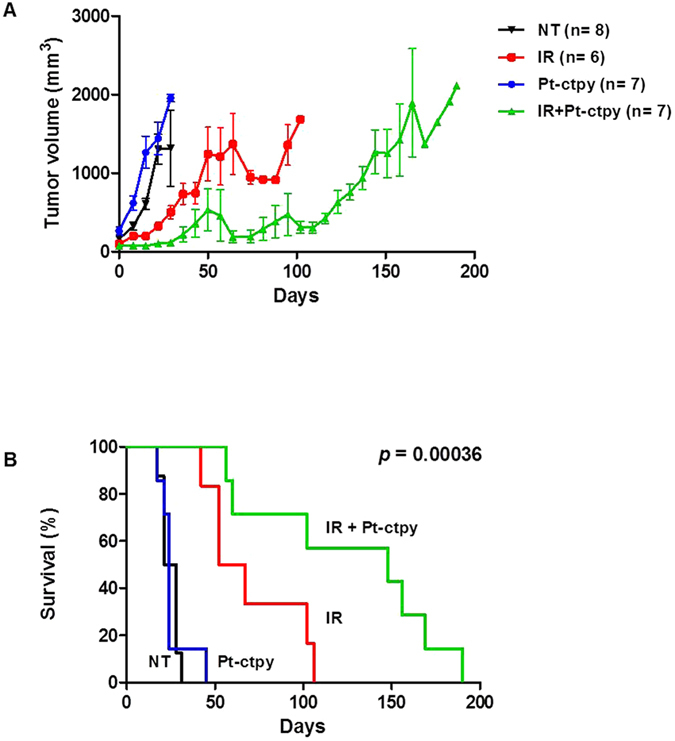
Antitumor efficacy of Pt-ctpy in combination with radiation on SF763 xenografts. GBM human xenografts with SF763 cells were done in the leg of nude mice. Pt-ctpy treatment was given intra- and peritumoraly at 2 mg/kg/day. In the combination experiments, Pt-ctpy was administered every 24 h for 5 days prior to single irradiation of 15 Gy and then 5 days after irradiation. (**A**) Representation of tumor growth kinetics in untreated group (NT) and in groups treated with radiation alone, Pt-ctpy alone or radiation + Pt-ctpy group. (**B**) Survival curves in these groups are significantly different.

Untreated animals exhibit a roughly exponential tumor growth with an overall survival of 24 days (Fig. 7A). In the group of recipient mice irradiated with a single dose of 15 Gy, we observed an inhibition of tumor growth during 30 days. In the group that received Pt-ctpy treatment alone, no tumor growth inhibition was observed. However, when Pt-ctpy treatment was combined with radiation, we noted a long tumor growth delay on average 90 days. Survival analysis showed a significant difference between the groups (Fig. 7B, *p* = 0.00036). In particular, survival of mice treated with Pt-ctpy in combination with radiation was significantly longer (median: 148 days) compared with those of animals treated with radiation alone (52 days) or Pt-ctpy alone (24 days) (*p* = 0.032 and *p* = 0.043, respectively). These data strongly suggest that the radiosensitizing effect of Pt-ctpy observed *in vitro* is transposable to *in vivo* conditions.

## Discussion

Pt-ctpy is a second-generation G4 ligand with a good affinity-selectivity ratio for G-quadruplex DNA as shown by FRET-melting and FID assays^20^. This compound belongs to the tolyterpyridine-metal complexes family known to interfere with quadruplex DNA both via stacking interaction on external G-quartets and via platination of the loop bases^17^,^18^. In this study, we found that submicromolar concentrations of Pt-ctpy (0.05 and 0.1 μM) reduced the proliferation of GBM and NSCLC cells in a concentration-dependent manner. Treated cells accumulated in the S-phase and GBM cells were also blocked in the G2/M-phase. In addition, the use of a higher Pt-ctpy concentration (0.5 μM) induced apoptosis, which is in line with the phenotypic profile previously described for cancer cells treated with a telomerase inhibitor^21^ or various G4 ligands such as RHSP4^22^, telomestatin^23^ and the bisquinolinium derivative 307A^24^. All together, these effects on cell cycle distribution and apoptosis can account for the decreased cell proliferation rate.

Pt-ctpy treatment modified the expression of telomere-related genes *hTERT* and *TRF1*. We observed an overexpression of *hTERT*, which might counteract the damaging effects of the stabilization of G4-DNA at telomeres through the extension of the 3′ single-stranded G-rich overhang^25^. We also noted a concomitant underexpression of *TRF1*, which controls access of telomerase to telomeric DNA^26^. These findings are in agreement with our previous results obtained with another G4 ligand, suggesting that they may represent compensatory mechanisms in response to treatment^16^ and/or a selection of cells with this expression profile during treatment.

As telomeric dysfunction was reported to increase radiosensitivity and decrease DNA repair capacity^4^, we investigated the impact of G4 ligands on the radiosensitization of GBM and NSCLC cells. Low, non-cytotoxic concentrations of Pt-ctpy showed a strong radiosensitizing effect on GBM and NSCLC cells. Radiation may alter telomeres directly, by inducing the damage of telomeric DNA, or indirectly, by telomere uncapping and alteration of telomere maintenance^27^. G4-ligand treatment leads to dysfunctional and unprotected telomeres, which are recognized as DSB^28^, and uncapped telomeres may interact with radiation-induced DSB^29^. Thus, telomere alterations induced by G4 ligands may interact with telomere damage and DSB induced by irradiation, resulting in enhanced radiosensitivity. Significant increases in DSB, TIF and telomere deletions observed in tumor cells after combined treatment with Pt-ctpy and radiation support this hypothesis. Of note, a greater sensitivity of GBM cells to radiation was associated with higher number of TIF using the G4-ligand RHPS4^30^. All these lesions may lead to early (apoptosis) or delayed (post mitotic) cell death, enhancing effects of irradiation.

Radiosensitizing effects of telomere dysfunction have previously been observed *in vivo*, using an animal model with telomerase deficiency^4^. In our mouse model with GBM tumor xenograft, we found that Pt-ctpy treatment enhanced antitumor effects of radiation. We note that Pt-ctpy treatment alone was well tolerated and no obvious toxicity was observed which is a basic prerequisite in view of clinical application.

In conclusion, we show that Pt-ctpy, a G-quadruplex ligand of the terpyridine-metal family, is able to enhance the sensitivity to ionizing radiation of GBM and NSCLC cell lines *in vitro*. This effect might result from induction of specific damages at DNA by the G4 ligand and in particular within telomeric regions although more in-depth mechanistic investigations are required to identify the molecular determinants of the radiosensitization. For instance, interferences with other quadruplex forming sequences like those found in *c-MYC* promoter as well as in promoters of other oncogenes cannot be excluded^18^ and may also contribute to the radiosensitization. Most importantly, our study provides the first evidence that a G-quadruplex-DNA interactive molecule is able to radiosensitize GBM xenografts. Although this radiosensitizing effect needs to be investigated *in vivo* with other human GBM and NSCLC xenografts, our study provides the basis for an innovative therapeutic approach using G-quadruplex ligands as radiosensitizers to improve the efficacy of radiation therapy without additional toxicity, especially in the case of human solid tumors characterized by a limited response to radiation alone.

## Material and Methods

### Cell lines

Human GBM cell lines SF763 and SF767 were kindly provided by Dr Moyal (UMR 1037 INSERM, Toulouse, France). Normal skin fibroblasts were obtained from a healthy 30-year-old female donor. Cells were cultured in DMEM medium with 10% of FCS, 1% of non-essential amino acids, 1% of sodium pyruvate and gentamicin (Invitrogen, Cergy-Pontoise, France). Human NSCLC A549 and H1299 cells were obtained from Grenoble University, UMR INSERM 823 (France) and were maintained in RMPI-1640 supplemented as indicated above. Cell lines were mycoplasma-free (Mycoplasma-Detection Kit, Invivogen, Toulouse, France).

### Chemical compound

**Figure 8:**
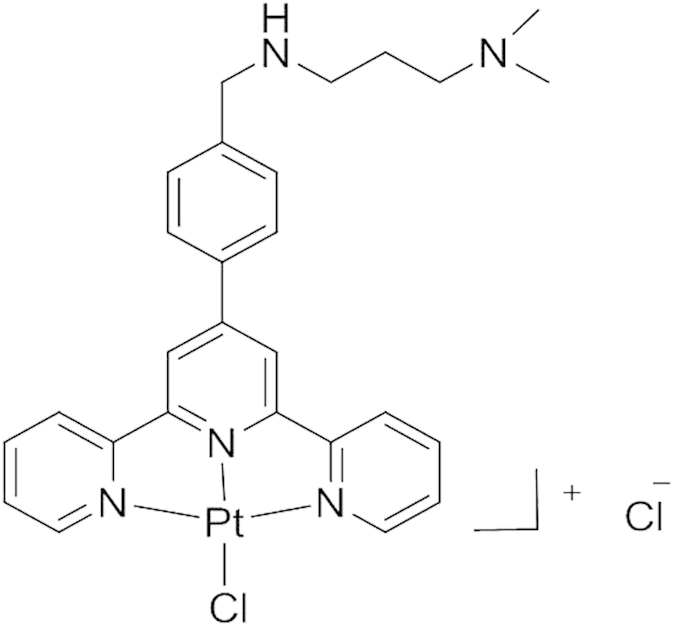
Chemical structure of Pt-ctpy.

The structure of a terpyridine-metal-organic complex Pt-ctpy is shown in Fig. 8. The synthesis and DNA-binding ability of this compound were described previously^20^. Pt-ctpy binds the telomeric quadruplex DNA with a ΔTm of 16–18 °C (FRET-melting assay) and an IC_50_ of 0.38 μM (G4-FID assay). Comparison of these data with the values obtained for the benchmark G4 ligand PhenDC3 (ΔTm = 30 °C, IC_50_ = 0.31 μM) indicates that Pt-ctpy exhibits high affinity for quadruplex conformation. This compound shows at least 20-fold better affinity for G-quadruplex DNA as compared to duplex DNA. Pt-ctpy displays good water solubility with a logP of 0.3 suitable for pharmacological applications.

### Proliferation assessment

Cells (50,000–100,000) were seeded in 25 cm^2^ culture flasks and incubated with 0.05–0.1 μM Pt-ctpy. Cells were replated once a week. The number of population doublings (PD) was calculated as follows: PD = log_2_(N_*f*_/N_*o*_), where N_*f*_ is the final cell number and N_*o*_ is the initial number of seeded cells.

### Cell cycle analysis

Cell membranes were lysed by immersion in liquid nitrogen. The cell pellet was then resuspended in 500 μL of ribonuclease A (Sigma, Saint-Quentin Fallavier, France) and 500 μL of propidium iodide (PI) (Sigma) for 20 min at room temperature. Cell cycle distribution and sub-G0/G1 fraction were determined by flow cytometry (EPICS XL; Beckman Coulter, Villepinte, France).

### Quantitative RT-PCR (RQ-PCR)

RNA was extracted using Trizol reagent and one μg of RNA was reverse transcribed with SuperScript II (Invitrogen). RQ-PCR was performed with the LightCycler system (Roche, Meylan, France). The primers used for amplification of *hTERT* and *TRF1* were described elsewhere^31^,^32^. *ABL* transcript was quantified for normalization^33^.

### Irradiation

Photon irradiations were performed at room temperature using a linear accelerator PHILIPS SL 75-5 (in X-ray production mode) at a dose rate of 3 Gray (Gy)/min for cell irradiation. For *in vivo* experiments, a single radiation dose of 15 Gy was delivered using two opposite 6 MV photon beams on the tumor-bearing limb.

### Clonogenic survival assay

Cells pre-treated with Pt-ctpy (0.05 or 0.1 μM) for one week were plated in 25-cm^2^ cell culture flasks and allowed to attach for 24 h. Cells were irradiated with a range of doses between 2 and 8 Gy and incubated with Pt-ctpy for 10 days, then fixed with methanol and stained with Giemsa. Colonies with >50 cells were scored. The surviving fraction at each dose was calculated relative to the plating efficiency of non-irradiated or non-treated control cells.

### 53BP1 immunofluorescence staining and telomere FISH

Cells were fixed with 4% paraformaldehyde, permeabilized with 0.5% Triton X-100 and incubated with a mouse monoclonal anti-53BP1 antibody (Santa Cruz Biotechnology, sc135748, Santa Cruz, CA, USA) overnight at 4 °C. Cells were then exposed to the secondary FITC-labeled chicken anti-mouse antibody (Invitrogen, A21022, Carlsbad, CA, USA). For telomere FISH on metaphase spreads, cells were hybridized with a Cy3-PNA [(CCCTAA)_3_, PN-TC050-005, Panagene, Daejeon, Korea) telomere probe. The nuclei were stained by 4′,6-diamidino-2-phenylindole. Fluorescence signals were analyzed using a Zeiss Confocal Laser Scanning microscope or a Zeiss Axioimager, Z1.

### *In vivo* experiments

All experiments involving animals were performed in accordance with protocols approved by the regional ethics committee (CEMEA Auvergne) in charge of animal experimentation (protocol no. CE3-09). NMRI female Nude (nu/nu) mice were purchased from Janvier Laboratories (Nantes, France). Mice were injected with SF763 cells at 10^7^ *sc* in the right lower limb. Treatments started when a tumor volume of 50 to 250 mm^3^ was evidenced. Pt-ctpy was delivered at 2 mg/kg/d inside and *s.c.* around the tumor for ten days (day 1 to 5 and 7 to 11). In combination experiments, Pt-ctpy was administered for five days (days 1–5) prior to a single irradiation with 15 Gy (at day 5) and then for additional five days after irradiation (days 7–11).

### Statistical analysis

Statistical differences between groups were determined by the *t*-test, Kruskal-Wallis H-test or ANOVA (analysis of variance) using the SEM software^34^. Survival time was defined as the time between the start of treatment and the date when the mice were euthanized. Kaplan-Meier survival curves were compared with the log-rank test.

## Additional Information

**How to cite this article**: Merle, P. *et al.* Highly efficient radiosensitization of human glioblastoma and lung cancer cells by a G-quadruplex DNA binding compound. *Sci. Rep.* **5**, 16255; doi: 10.1038/srep16255 (2015).

## Supplementary Material

Supplementary Information
