# Supplementary material for: Highly efficient radiosensitization of human glioblastoma and lung cancer cells by a G-quadruplex DNA binding compound
Source: Sci Rep. 2015 Nov 6;5:16255. doi: 10.1038/srep16255 (PMC4635363; doi:10.1038/srep16255)
Supplement: Supplementary Information [file srep16255-s1.pdf]

Highly efficient radiosensitization of human glioblastoma and lung cancer cells by a G-quadruplex DNA binding compound

Patrick Merle, Marine Gueugneau, Marie-Paule Teulade-Fichou, Mélanie Müller-Barthélémy, Simon Amiard, Emmanuel Chautard, Corinne Guetta, Véronique Dedieu, Yves Communal, Jean-Louis Mergny, Maria Gallego, Charles White, Pierre Verrelle, Andreï Tchirkov

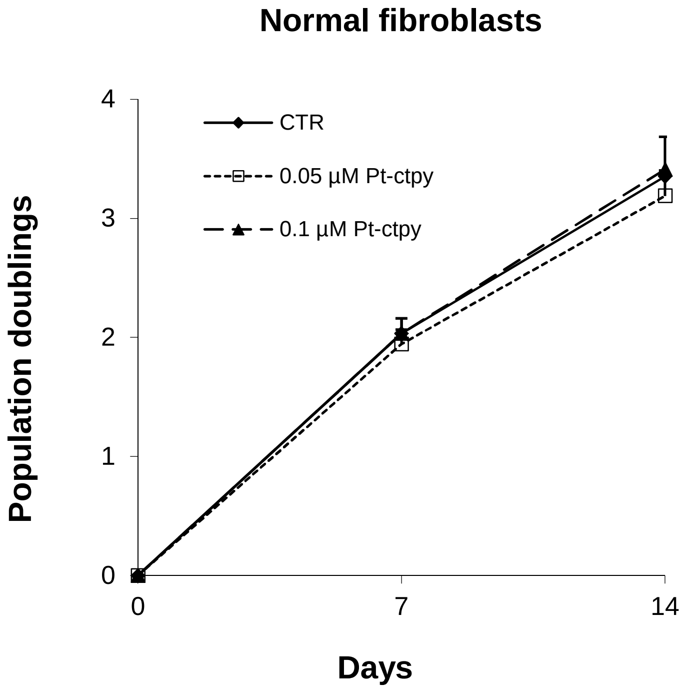

Supplementary Figure 1. Normal human fibroblast cultures were treated continuously with Pt-ctpy (0.05 and 1 µM) for 14 days as compared with non-treated cells (NT). No proliferation inhibition was found in normal fibroblasts. Analysis of cellular viability after 7 and 14 days indicated that the treatments were not toxic (<10% of cells colored with trypan blue).
